# Role of hormonal and inflammatory alterations in obesity-related reproductive dysfunction at the level of the hypothalamic-pituitary-ovarian axis

**DOI:** 10.1186/s12958-018-0366-6

**Published:** 2018-05-09

**Authors:** Michelle Goldsammler, Zaher Merhi, Erkan Buyuk

**Affiliations:** 1 Montefiore’s Institute for Reproductive Medicine and Health, Department of Obstetrics & Gynecology and Women’s Health, Albert Einstein College of Medicine, Montefiore Medical Center, Hartsdale, NY USA; 2 Department of Obstetrics and Gynecology, Division of Reproductive Biology, NYU School of Medicine, New York, NY USA; 3 Department of Biochemistry, Albert Einstein College of Medicine, Bronx, NY USA

**Keywords:** Obesity, Ovary, HPO, Advanced glycation end products, Monocyte chemotactic protein-1

## Abstract

**Background:**

Besides being a risk factor for multiple metabolic disorders, obesity could affect female reproduction. While increased adiposity is associated with hormonal changes that could disrupt the function of the hypothalamus and the pituitary, compelling data suggest that obesity-related hormonal and inflammatory changes could directly impact ovarian function.

**Objective:**

To review the available data related to the mechanisms by which obesity, and its associated hormonal and inflammatory changes, could affect the female reproductive function with a focus on the hypothalamic-pituitary-ovarian (HPO) axis.

**Methods:**

PubMed database search for publications in English language until October 2017 pertaining to obesity and female reproductive function was performed.

**Results:**

The obesity-related changes in hormone levels, in particular leptin, adiponectin, ghrelin, neuropeptide Y and agouti-related protein, are associated with reproductive dysfunction at both the hypothalamic-pituitary and the ovarian levels. The pro-inflammatory molecules advanced glycation end products (AGEs) and monocyte chemotactic protein-1 (MCP-1) are emerging as relatively new players in the pathophysiology of obesity-related ovarian dysfunction.

**Conclusion:**

There is an intricate crosstalk between the adipose tissue and the inflammatory system with the HPO axis function. Understanding the mechanisms behind this crosstalk could lead to potential therapies for the common obesity-related reproductive dysfunction.

## Background

According to a recent population study, approximately 39% of the population over the age of 20 and 18% of children between the ages of 2–19 are obese [1]. Obesity causes a huge economic burden where it is estimated that obesity will add 48–66 billion dollars in related health care expenditures by the year 2030 [2]. This is due to obesity-related comorbidities such as diabetes mellitus, hypertension, dyslipidemia, and cardiovascular disease [3]. Besides these chronic disorders, obesity is also associated with reproductive and obstetric complications such as menstrual irregularities, subfertility, endometrial hyperplasia and cancer, as well as poor obstetrical and perinatal outcomes [4–7]. This review will focus on the relationship between obesity and female reproductive function with a focus on alterations in the hypothalamic pituitary ovarian (HPO) axis and the direct effect of obesity-related inflammatory processes on ovarian function.

### Normal HPO Axis

The female reproductive physiology is a complex interaction between neuroendocrine and endocrine signaling affecting the hypothalamus, the pituitary gland and the ovaries. At the level of the hypothalamus, gonadotropin releasing hormone (GnRH) pulses activate the pituitary release of the two gonadotropins, follicle-stimulating hormone (FSH) and luteinizing hormone (LH) [8, 9]. FSH and LH in turn act on the ovaries to stimulate follicular growth and result in the production of estradiol and, following ovulation, progesterone [10]. Estradiol, together with ovarian inhibin B, acts primarily in a negative feedback loop on the hypothalamus suppressing the release of FSH. When estradiol reaches a sustained threshold in concentration and duration, for at least 24–48 h [11–13], it provides a positive feedback that increases the frequency and decreases the amplitude of GnRH pulses thereby activating increased pituitary release of LH surge for ovulation to occur [14]. There are multiple regulators of this cycle and our review is limited to those regulators, which are altered, directly or indirectly, in overweight and obese women.

The HPO axis plays a crucial role in pubertal transition. Normal pubertal development is under two main controls, adrenal and hypothalamic [15]. The adrenal glands participate in adrenarche, which often precedes the remainder of pubertal events. Adrenarche is independent of the hypothalamic pubertal changes as observed in several disorders of sexual differentiation [16]. The hypothalamus, with its GnRH pulse generator responsible for the initiation of puberty, is quiescent in childhood, likely under GABAergic (gamma-aminobutyric acid) inhibitory control [17, 18]. Normal central activation of the hypothalamus via kisspeptin and glutamate neurons in the arcuate nucleus results in nocturnal increases in low frequency LH pulses [19]. These pulses become more prevalent throughout the day until the GnRH pulse generator achieves the frequency and amplitude sufficient for cyclic ovarian hormonal function and ultimately menarche. During normal puberty, there is a physiologic insulin resistance that helps achieve the anticipated pubertal weight gain and growth [20–22]. The insulin resistance leads to decreased hepatic derived circulating sex hormone-binding globulin (SHBG), which in turn increases circulating free estradiol levels. This may also contribute to a parallel adrenal activation resulting in more androgen production and ultimately adrenarche [17, 18]. Increasing estradiol levels, in turn, are significantly associated with growth velocity, [23] possibly through the stimulation of the growth hormone – Insulin like growth factor 1 (IGF-1) axis [24, 25].

### Obesity and the HPO axis

It has been thought that obesity changes the expected time course of puberty and leads to earlier thelarche, adrenarche and menarche [26–28]. Adiposity, and specifically the distribution of the adipose tissue, contributes to fluctuations in peripheral steroid hormone secretion, thereby impacting pubertal development [29]. For instance, the adipocytes contain aromatase, that both assists in the production [30] and the conversion of steroid hormones, mainly androgens to estrogens. Population studies have demonstrated a trend for earlier menarche, due to the obesity epidemic, over the past 30 years [31], highlighting the potential for significant increase in health care risks and cost for these young girls. Increased adipose tissue quantity rather than increased sensitization or increased aromatase activity during aging [32] and therefore increased peripheral production of estrogens leads to increased rate of serious hormone dependent cancers, such as endometrial and breast cancer [33, 34].

Obesity decreases pituitary LH pulse amplitude and mean LH release without changing its frequency, leading to impaired luteal phase [35]. Additionally, obesity may affect various components of the HPO axis, and may have direct effect on ovarian function independent of hypothalamic pituitary function. We will elucidate in the following sections the changes that occur in the metabolic and inflammatory systems that are related to ovarian dysfunction.

#### Leptin

Leptin is a 16-kDa adipokine secreted primarily by white adipose tissue, although it can be secreted from other tissues, such as gastric mucosa [36]. Serum leptin levels positively correlate with the amount of adipose tissue in the body [37]. Leptin has several functions, which in concert reflect body energy homeostasis. It acts to stimulate energy expenditure and suppresses appetite via its signaling at the level of the hypothalamus [18, 38]. These findings were confirmed with mouse knockout model where leptin receptor knockout mice become obese with a rapid and persistent weight gain [39]. Because leptin suppresses appetite, it was studied as a potential target for weight loss drug development [40]. However, it has been shown that increased exogenous leptin does not necessarily induce weight loss [41, 42] suggesting that there is a resistance state to the action of leptin at the level of the hypothalamus. Indeed, high circulating leptin levels observed in obese women supports the concept of leptin resistance [37]. This desensitization to leptin is not a complete blockage of leptin signaling and can possibly be reversed with weight loss [43].

Leptin’s energy homeostatic function in part contributes to its effect on puberty. Puberty requires a certain energy balance to proceed [22, 44]. Leptin has a permissive action for pubertal progression, but it is not the initiator or the sole mechanism by which puberty occurs [45]. Conversely, hyperleptinemia may initially cause earlier reproductive maturation, and prolonged high leptin levels could lead to ovulatory dysfunction [46]. On the other hand, low leptin levels observed in energy-deficient states are associated with delayed or lack of puberty, when puberty is not physiologically desired [47].

Leptin affects GnRH pulse neurons indirectly though GABA or kisspeptin [39, 48]. Both GABA and kisspeptin neurons have a focus in the arcuate nucleus, where leptin receptors are readily available [39, 49, 50]. Knockout models for the leptin receptor that is found on GABA specific neurons display delayed puberty and decreased fecundability in animal models [39]. Similarly, loss of function gene mutation in the kisspeptin gene results in delayed or absent puberty [51]. Hypothalamic kisspeptin levels often correlate with leptin levels [50] in line with low energy states, making this an alternate pathway for leptin signaling [52]. Both low serum, and therefore hypothalamic, leptin levels and low hypothalamic kisspeptin levels decrease LH secretion by the pituitary, ultimately affecting ovulation [52].

Studies on the effect of leptin on ovarian folliculogenesis and ovulation have shown conflicting results. Leptin has been isolated in both mural and cumulus granulosa cells and within the follicular fluid of pre-ovulatory follicles in women undergoing in vitro fertilization (IVF) [53]. Serum leptin levels increase after ovulation and have peak levels in the mid-secretory phase, possibly contributing to the implantation window within the endometrium [54]. Some studies suggested an inhibitory effect of leptin, especially on early follicular development, while others suggested that leptin could induce ovulation, independent of LH [55] since there seems to be an interaction between estradiol and leptin [55, 56]. In a mouse model, leptin administration increased LH levels and follicular development corresponding to an increase in ovarian tissue weight [57]. In addition, IVF outcome studies pointed to leptin as negatively correlated to reproductive outcome. In one study of serum leptin levels in patients undergoing IVF, higher leptin:BMI ratio was associated with a decreased number of good quality embryos and lower implantation and pregnancy rates [58]. At the ovarian level [46], supraphysiologic levels of leptin inhibits androstenedione and progesterone production [59, 60]. Additionally, human granulosa and cumulus cells exposed to leptin in vitro lead to a downregulation in anti-Müllerian hormone (AMH) gene expression via the JAK/STAT pathway [61] potentially leading to ovulatory dysfunction. In summary, normal leptin homeostasis is required for normal physiologic functions at the level of the hypothalamus and the ovaries. While low levels of leptin could disrupt the GnRH pulsatility, supraphysiologic levels of leptin could disrupt ovarian folliculogenesis [46, 55, 62].

#### Ghrelin

Ghrelin is an orexigenic enterokine composed of 28 amino acids related to the oxyntomodulin family of intestinal peptides [63]. It is predominantly produced by the stomach, but is also detectable in many other tissues, like bowel, pancreas, hypothalamus and pituitary [64]. Ghrelin is increased in fasting states and stimulates appetite to compensate for decreased nutritional intake [65]. Mouse models confirm that ghrelin increases food consumption as an immediate and short-term effect [66] by acting on the arcuate nucleus in the hypothalamus and stimulates appetite via neuropeptide Y (NPY) and agouti related protein (AgRP) neurons [67]. One of ghrelin’s targets is the hypothalamic arcuate nucleus where it increases the expression of NPY/AgRP mRNA [68] resulting in increased body weight and inhibiting proopiomelanocortin (POMC) neurons [69]. Double knockout (NPY-/AgRP-) mouse models, or other NPY/AgRP deficient models demonstrated suppression of ghrelin-induced appetite stimulation as compared to wild type control animals. Deficiency of both NPY and AgRP is necessary to abolish the effect of ghrelin while knockout of only one of these mediators is not sufficient to suppress ghrelin’s effect [66].

In the hypothalamus, ghrelin can decrease both GnRH secretion and pulsatility [70–73] possibly through NPY- and AgRP- mediated mechanisms [74]. Vulliémoz et al. suggested that this effect is part of the homeostatic control of reproductive function in response to nutritional changes. In low energy states, such as fasting, ghrelin levels are increased, thereby altering the HPO axis cyclic activity [73]. Ghrelin also stimulates other pituitary hormone release, such as adrenocorticotropic hormone and prolactin [75]. In low energy states where ghrelin levels are elevated, prolactin levels may be increased potentially leading to disruption of ovarian cyclicity. Ghrelin has not clearly been shown to have a direct effect on pubertal transition. However, ghrelin levels decrease throughout childhood as puberty approaches [75]. Given its inverse relationship to adiposity, decreasing ghrelin levels may be a reflection of the increasing weight gain observed in the pre-pubertal stage [76].

Studies on the effect of ghrelin on ovarian steroidogenesis have shown mixed results [74, 77]. Animal studies suggest that ghrelin could induce estradiol and/or progesterone production, [78] or could inhibit estradiol release via inhibiting CYP19A1 (aromatase enzyme) expression [74, 79, 80]. These effects may be dose-dependent [81] or related to the activated portion of the ghrelin molecule [79]. The full-length ghrelin molecule stimulates estradiol secretion while certain amino acid fragments of ghrelin are inhibitory for estradiol secretion [79]. Ghrelin could affect folliculogenesis by increasing cell proliferation and decreasing apoptosis and follicular atresia [82, 83]. Similarly, chronic ghrelin infusion leads to increase in follicle number and decrease in corpus luteum number in a rat model [84]. Additionally, ghrelin, with its connection to obesity, is related to insulin regulation and insulin resistance where it decreases insulin secretion and sensitivity [64, 85, 86] ultimately leading to insulin resistance [87].

Thus, these findings suggest that ghrelin may indirectly contribute to puberty through general energy homeostasis such as insulin action, and it acts at the level of the hypothalamus, pituitary and ovary leading to alterations in normal reproductive function.

#### Neuropeptide Y and Agouti-related protein: (NPY/AgRP)

The connection between peripherally circulating adipokines, enterokines and the HPO axis centers on a collection of neurons within the arcuate nucleus, which secrete the neuropeptides NPY and AgRP [88]. NPY is a 36 amino acid neurotransmitter peptide predominantly expressed in sympathetic neurons. It stimulates fat angiogenesis and proliferation via both a central hypothalamic and peripheral mechanism [49, 89]. AgRP is a 112 amino acid neuropeptide expressed in the arcuate nucleus [90, 91] and stimulates appetite [91]. Both NPY and AgRP are orexigenic neurons and interact with ghrelin to promote appetite [66].

Under normal physiologic conditions, NPY concentration increases in the portal capillary blood during the ovulatory surge to potentiate the action of GnRH on pituitary gonadotropin secretion [92, 93]. Similarly, AgRP engages the GnRH pulse generator neurons within the hypothalamus and regulates gonadotropin release. Both NPY and AgRP have tonic inhibitory effects where they could be stimulated by ghrelin in order to decrease GnRH pulse frequency and amplitude [94]. Infusion of NPY, independent of ghrelin, also decreases pituitary LH secretion [95]. NPY could have a negative regulatory impact on ovarian folliculogenesis where it could have a pro-apoptotic and an anti-proliferative effect [96]. NPY has been shown to have no effect on progesterone secretion [97], but it recently has been found to stimulate estradiol and testosterone secretion in catfish [98]. Data pertaining to the direct effect of NPY and AgRP on ovarian steroidogenesis and folliculogenesis are still limited and further studies are required.

#### Adiponectin

Adiponectin is a 30-kDa adipokine secreted by the adipose tissue [99]. Opposite to leptin, adiponectin levels increase with starvation [99]. Adiponectin acts in the brain by binding to the adiponectin receptor 1 (AdipoR1) in the arcuate nucleus. This binding leads to activation of AMPK resulting in increased food intake and reduced energy expenditure [100]. Adiponectin production increases insulin sensitivity and is inversely correlated with adiposity [101]. Knockout studies demonstrated that the absence of adiponectin causes severe insulin resistance that is reversible with administration of exogenous adiponectin [102]. Low adiponectin levels, as seen in certain genetic polymorphisms, have been linked to insulin resistance, metabolic syndrome and type 2 diabetes mellitus [103–105]. Interestingly, the adiponectin gene is located at 3q27, near the diabetes susceptibility gene locus [103, 106]. Adiponectin is structurally similar to the pro-inflammatory TNF- α family, however it functions as an anti-inflammatory adipokine, inhibiting the production of TNF-α within the adipocyte [102]. Conversely, pro-inflammatory agents such as TNF-α, IL-6 and IL-8 are implicated in the development of insulin resistance via hypothalamic inflammation [107]. This inflammation in turn contributes to both insulin and leptin resistance, promoting further obesity and subsequently diabetes [107]. In fact, in obese women adiponectin levels are low and pro-inflammatory markers, such as TNF-α, IL-6, and CRP are increased [46].

Adiponectin and its receptors are found in many organs including the ovaries [108–111]. Adiponectin acts in concert with insulin and IGF-1 to mediate changes within the granulosa cells during the periovulatory phase. Through IGF-1, it increases ovarian production of estradiol and progesterone in rat ovaries [108] possibly by up-regulating StAR gene [109]. It also causes vasodilation with the upregulation of VEGF and COX-2 expression in the periovulatory ovary [109]. Adiponectin knockout mice show ovarian dysfunction reflected by fewer oocytes, more atretic follicles, prolonged diestrus cycles and decreased LH receptor activity [112]. In a retrospective case-controlled analysis, adiponectin levels were higher in women who conceived after IVF and positively correlated with the number of oocytes retrieved, independent of BMI [113]. Similarly, while adiponectin levels are low or minimal in human and mouse granulosa cells, its presence enhances fertilization rates and embryo development [114, 115].

These findings suggest that adiponectin is notable for its action in mediating insulin sensitivity, with its receptors found at every level of the reproductive axis making it a great therapeutic target for ovulatory dysfunction.

#### Insulin

Insulin is a 51 amino acid protein synthesized in the beta islet cells of the pancreas. Its release is stimulated by glucose in the gastrointestinal tract from ingestion, as well as various amino acids directly [116]. Insulin levels rise and its sensitivity decreases with obesity [116]. Insulin resistance, in conjunction with obesity, impacts reproduction. While not a direct adipokine, adipose tissue stimulates pancreatic beta islet cells to release insulin. Hyperinsulinemia acts on the liver to cause a decrease in SHBG production [117]. This in turn leads to increased free circulating steroid hormone levels, such as estrogens and androgens. Insulin increases androgen production by two independent pathways; first by up-regulating CYP17A1 enzymes, which increase androgen production in both the adrenal gland and the ovary [118]. Second, insulin augments LH action on the ovary to increase androgen production and secretion [118, 119]. Insulin resistance is associated with higher leptin levels [38]. As noted previously, higher circulating leptin levels lead to leptin resistance which in turn leads to greater insulin resistance.

Insulin acts on the pituitary to modulate the GnRH receptor and increases LH secretion after GnRH stimulation [120]. Insulin augments FSH activity by increasing ovarian steroidogenesis and inducing LH responsiveness [121]. Hyperinsulinemia is consequently associated with elevated basal LH levels and hyperandrogenism [119]. Insulin alone does not have an effect on ovarian response to gonadotropic hyperstimulation during IVF in women without underlying insulin resistance [122]. Rather, the changes seen during IVF stimulation are due to insulin resistance [122]. Syndromic severe insulin resistance, as seen in some genetic disorders, is associated with enlarged ovaries and hyperandrogenism independent of gonadotropin levels. Elevated insulin levels over a long period of time can lead to increased autophosphorylation of its receptor, which can inactivate one of its downstream transducers, GSK3. This inactivation can lead to spindle disruption within growing oocytes [123, 124]. Additionally high insulin levels during oocyte development disrupt chromatin remodeling within mouse oocytes, thereby contributing to poorer oocyte quality [123]. Mouse models show that the pituitary is still sensitive to changes in insulin levels despite peripheral insulin resistance and basal hyperinsulinemia [120].

Disruption of insulin signaling in diet-induced obesity improves reproductive cyclicity in mice, suggesting that insulin represents a mediator for pituitary LH dysregulation in obesity [120]. Further study of insulin at the level of the ovary, specifically in insulin receptor knockout mice in theca cells, also demonstrates improved cyclic reproduction in mice, showing a coordinated effect of insulin along the HPO axis to disrupt cyclicity but not pubertal onset [118]. In summary, insulin’s action is known to be necessary for changes in food intake and body weight. Its well-studied actions on the HPO axis and its relationship to adipokines such as leptin and adiponectin makes it a major player in female reproduction.

#### AGEs and MCP-1

Obesity is a state of chronic inflammation with macrophage infiltration into various tissues. Macrophage infiltration into adipose tissue is directly correlated to both the degree of adiposity as well as chemokine/adipokine production, such as MCP-1 [125]. The pattern of macrophage infiltration is similar to that found in disorders associated with chronic inflammation such as rheumatoid arthritis [125]. In addition to elevated circulating inflammatory markers, such as TNF-α, IL-6, and CRP [46], obese women have elevated levels of circulating AGEs [126] and MCP-1 [127]. In animal studies, obese mice have higher MCP-1 levels, which correlate with insulin resistance [128].

The pro-inflammatory AGEs may be part of the link between diet-induced obesity and inflammation, with AGEs inducing MCP-1 gene expression [129] thus forming the AGEs/MCP-1 axis. AGEs are highly reactive molecules formed by non-enzymatic cross-linking of proteins, lipids and nucleic acids with glucose [130, 131]. They may be formed endogenously or exogenously ingested as part of diet or through smoking [132]. AGE levels are elevated in chronic diseases such as type 2 Diabetes Mellitus, metabolic syndrome, cardiovascular disease, and neurodegenerative disorders [133–135]. AGEs have also been studied for their effect on reproduction [135–137].

Kandaraki et al. has demonstrated in human immortalized granulosa cells (KGN cell line) that AGEs attenuate LH- and FSH-induced ERK signaling needed for cell proliferation and proper follicular growth [138], one possible mechanism for AGE-induced ovulatory dysfunction. Similarly, AGEs interfere with glucose transport within granulosa cells [136]. Our recent data have shown that high-AGE diet could induce ovulatory dysfunction in a mouse model, as reflected by prolonged diestrus phase (unpublished data). We have also shown that high-fat diet induced obesity leads to ovulatory dysfunction in mice, as demonstrated by fewer oocytes ovulated following superovulation compared to mice on normal chow diet (controls) [139]. This ovarian dysfunction was not observed in MCP-1 knockout mice that became obese following ingestion of a high-fat diet, suggesting that lack of MCP-1 may be protective against high-fat- and obesity-induced ovarian dysfunction. Further supporting this hypothesis, we showed that elevated serum MCP-1 levels were associated with poorer outcome in women undergoing IVF [140], an effect that is pronounced in women with already diminished ovarian reserve. Additionally, AGE levels in follicular fluid were negatively correlated with IVF outcome parameters: fewer oocytes retrieved and fertilized, fewer embryos and lower ongoing pregnancy rate [137]. Taken together, these observations suggest that obesity may have direct deleterious effects on the ovaries partly through activation of inflammatory AGE/MCP-1 axis.

### Relationship between obesity and assisted reproductive technology (ART) outcome

Population studies on the clinical sequelae of obesity provide a connection between obesity and subfertility. Several studies indicated that obesity is a risk factor for ovulatory dysfunction [141–143]. For women who ovulate regularly, obesity increases the time to conception; for instance, a high waist- height ratio decreases fecundity by 30% [144]. Van der Steeg et al. found that in ovulatory infertile women (who underwent fertility evaluation but did not yet receive treatment), for every BMI unit over 29, there was a 5% decrease in the probability of a conception [145]. This increased time to conception is not only in the infertile population. Even in obese fertile women there was an increased time to conception from 3 to 5 months [146]. Other population studies have shown that 33% of obese women at age 23 did not conceive spontaneously when trying to conceive for 12 months [147]. This increases the number of couples meeting criteria for infertility and therefore for potential ART interventions [147]. Obesity could also impact ovarian reserve markers. Studies have shown that obesity is negatively correlated with serum AMH [148, 149], FSH, LH and inhibin B levels [150]. These markers provide added support for a clinician’s analysis of a couple’s fertility potential and may guide treatment options [151].

Obesity does not only affect spontaneous pregnancy rates in fertile patients, but it also confers a risk for poorer ART outcome. While earlier data could not demonstrate adequate convincing evidence of poorer ART outcome parameters, recent data supports this correlation. Large national cohort studies as well as systemic reviews and meta-analyses suggest that increasing BMI is negatively correlated with implantation, clinical pregnancy, and live birth rates [152–154]. Obese patients require higher doses of gonadotropins but achieve lower serum estradiol levels and lower number of oocytes retrieved [152, 155]. The oocytes of obese women tend to be smaller with decreased fertilization potential, leading to a decreased blastocyst formation and decreased trophectoderm cell number [156]. Additionally, obese women have higher cycle cancellation rates, possibly due to changes in pharmacodynamics of GnRH antagonists (clinically used to inhibit ovulation) leading to early LH surge and premature ovulation [157]. While not all studies identified these specific adverse ART outcome measures (i.e. smaller oocytes, decreased embryo quantity and quality), they still demonstrated a lower clinical pregnancy and live birth rates-- up to 50% decrease compared to control women with normal BMI [152, 158].

The poorer IVF outcomes observed in obese patients are quite intriguing. These observations suggest that the effect of obesity on ovarian function is not solely dependent on the HPO axis, since gonadotropins are supplied exogenously during IVF cycles, thus bypassing the HPO axis. Obesity adversely impacts ART outcome differently in different ethnic populations [159–161]. However, these findings were inconsistent in the literature [162–164] and may actually be in part due to ethnic variations in BMI rather than difference in ethnicity itself [159, 161].

Obesity could disrupt endometrial receptivity leading to poorer implantation rates [158, 165, 166], arguably due to endometrial inflammation. Inflammatory markers such as IL-6 and TNF-α have been implicated in lower implantation rates [46]. Similar to macrophage infiltration in the adipose tissue [125], we have shown that there is increased expression of macrophage markers in the ovaries of obese mice following the ingestion of a high-fat diet [167]. Moreover, mice given high-fat diet ovulated fewer oocytes following superovulation, further supporting the notion that obesity may have direct effect on ovaries, independent of HPO axis. Clearly the data to date demonstrates that obesity affects ART outcome in women undergoing IVF possibly via actions on all aspects: oocyte, embryo and endometrium.

## Conclusion

With the uncurbed obesity epidemic, more reproductive-aged women will face metabolic and reproductive complications. Body energy hemostasis is closely linked to reproductive function through many hormones, adipokines, cytokines, and growth factors that act at the level of the brain and the ovaries. Obesity is also a state of chronic inflammation, which may directly affect ovarian function possibly by increased macrophage infiltration in the ovaries through MCP-1 mediated pathways. The elevation of AGEs in the serum and tissues of obese women may exacerbate the reproductive dysfunction associated with adiposity and may provide, along with MCP-1, a crucial link between obesity and ovarian macrophage infiltration. Each of these molecules and their prospective pathways may represent potential therapeutic targets in order to improve the overall reproductive health of overweight/obese women. Obesity, with its alterations in the AGEs/MCP-1 axis, could disrupt the ovarian microenvironment potentially compromising oocyte competence, formation of healthy embryos and ultimately conception.

Finally, this review underscores a critical need to uncover the mechanistic actions of molecules that affect almost every level of the HPO axis. Obesity, a significant and growing public health problem, is an overwhelming condition that causes reproductive disturbances in women in part due to ovarian dysfunction. Losing weight is commonly challenging and often not sustainable. Thus there is a need to establish therapies for ovarian dysfunction and to improve ovarian health in the obese patient population.
